# Dengue virus infection among long-term travelers from the Netherlands: A prospective study, 2008-2011

**DOI:** 10.1371/journal.pone.0192193

**Published:** 2018-02-07

**Authors:** Femke W. Overbosch, Janke Schinkel, Ineke G. Stolte, Maria Prins, Gerard J. B. Sonder

**Affiliations:** 1 Department of Infectious Diseases, Public Health Service (GGD), Amsterdam, the Netherlands; 2 National Coordination Centre for Traveller’s Health Advice (LCR), Amsterdam, the Netherlands; 3 Department of Medical Microbiology, Laboratory of Clinical Virology, Academic Medical Center, Amsterdam, the Netherlands; 4 Department of Infectious Diseases Research and Prevention, Public Health Service (GGD), Amsterdam, the Netherlands; 5 Department of Internal Medicine, Division of Infectious Diseases, Amsterdam Infection & Immunity Institute (AI&II), Academic Medical Center, Amsterdam, the Netherlands; 6 Department of Internal Medicine, Division of Infectious Diseases, Tropical Medicine and AIDS, Academic Medical Center, Amsterdam, the Netherlands; Institut Pasteur, FRANCE

## Abstract

**Background:**

Dengue is increasing rapidly in endemic regions. Data on incidence among travelers to these areas are limited. Five prospective studies have been performed thus far, mainly among short-term travelers.

**Objective:**

To obtain the attack and incidence rate (AR, IR) of dengue virus (DENV) infection among long-term travelers and identify associated risk factors.

**Methods:**

A prospective study was performed among long-term travelers (12–52 weeks) attending the Public Health Service in Amsterdam. Clients planning to travel to (sub)tropical countries were invited to participate. Participants kept a travel diary, recording itinerary, symptoms, and physician visits. Pre- and post-travel blood samples were serologically tested for the presence of Anti-DENV IgG antibodies. Seroconversion was considered suggestive of a primary DENV infection. Anti-DENV IgG present in both corresponding samples in combination with a post-/pre-travel ratio of ≥4:1 was suggestive of a secondary infection. Risk factors for a DENV infection were studied using poisson regression.

**Results:**

In total, 600 participants were included; median age was 25 years (IQR: 23–29), 35.5% were male, and median travel duration was 20 weeks (IQR: 15–25). In 39 of 600 participants (AR: 6.5%; 95% CI 4.5–8.5%) anti-DENV IgG test results were suggestive of a recent infection, yielding an IR of 13.9 per 1,000 person-months traveling (95%CI: 9.9–19.1). No secondary infections were found. IR for Asia, Africa, and America were comparable and 13.5, 15.8, and 13.6 per 1,000 person-months respectively. Of participants with a recent DENV infection, 51% did not report dengue-like illness (DLI) or fever, but 10% were hospitalized. In multivariable analysis, travelers who seroconverted were significantly more likely to be vaccinated with ≥2 flavivirus vaccines for the current trip or to have reported DLI in >1 consecutive weeks.

**Conclusions:**

Long-term travelers are at substantial risk of DENV infection. Half of those with a DENV infection reported no symptoms, but 10% were hospitalized, demonstrating the importance of advising anti-mosquito measures during travel.

## Introduction

Dengue is an arthropod-borne viral disease found in (sub)tropical regions and is endemic in more than 100 countries in Africa, the Americas, the Eastern Mediterranean, South East Asia, and the Western Pacific. The disease is transmitted by Aedes mosquitoes, and caused by four serotypes of dengue virus (DENV-1, -2, -3, and -4) [1]. An estimated 3.9 billion people worldwide are at risk [1]. Infection with a particular serotype provides lifelong immunity against that serotype after recovery, but cross-immunity to the other serotypes is only partial and temporary [1]. Infection is often asymptomatic or subclinical, but can produce a wide range of illness in which symptoms vary from a mild febrile self-limiting illness to a severe disease [2]. An estimated 390 million infections occur per year [3]. Individual risk factors determine the severity of disease and include age (such as infants with a primary infection born to dengue-immune mothers or children with a secondary dengue infection), ethnicity (white individuals), certain chronic diseases, and secondary DENV infection with a different serotype [2]. No specific curative treatment is available and although DENV vaccines have been developed, they are not yet available for travelers [4].

Transmission in endemic areas has increased and the proportionate morbidity of DENV among travelers returning ill has risen [5]. In endemic areas, predominantly in (semi-)urban settings, the increase is related to demographic and societal changes, such as the unprecedented population growth and uncontrolled urbanization of the past 50–60 years [2]. The worldwide incidence has increased 30-fold, though it is not clear if this is related to the increase among travelers.

Clinical surveillance studies are not suitable to calculate the risk of DENV infections among travelers, as they disregard asymptomatic and subclinical DENV infections, and do not include data regarding numbers of travelers [6]. Only five prospective studies performed in the last two decades calculated incidence rates of all DENV infections in cohorts of travelers to endemic countries, of which four predominantly focused on short-term travelers [7–10] and one on participants who traveled 3–6 months [11].

To gain more insight into the risk of DENV infection for long-term travelers, we prospectively studied Dutch long-term travelers who traveled ≥12 and ≤52 weeks to (sub)tropical countries. We estimated the prevalence (P) before traveling and the attack rate (AR) and incidence rate (IR) of DENV infections during travel. In addition, we identified risk factors associated with DENV infection.

## Methods

### Study population

A prospective mono-center study of immunocompetent Dutch travelers was conducted at the Public Health Service travel clinic in Amsterdam from December 2008 to September 2011. All clients aged ≥18 years planning to travel to any (sub)tropical country in Sub-Saharan Africa, Central America, the Caribbean, South America, or Asia for ≥12 and ≤52 weeks were invited to participate. All participants were seen by a medical doctor or nurse specialized in travel medicine and were advised according to Dutch National Guidelines on Traveler’s Health Advice [12], including oral and written information about how to avoid mosquito-borne infections.

### Ethics statement

The study protocol was approved by the Medical Ethics Committee of the Academic Medical Center of Amsterdam (MEC 08/064). Participants were included after obtaining written informed consent.

### Study procedures

A standardized questionnaire in Dutch or English was used to collect data before departure on socio-demographics, travel history, vaccination status, and purpose of travel (tourism, work/education, visits to friends and/or relatives). Participants were given a digital thermometer (Huikeshoven Medical, Tiel, the Netherlands) and asked to take their temperature if they felt feverish. They were also asked to keep a structured, weekly travel diary, recording their itinerary, signs of disease, use of insect repellent containing N,N-diethyl-meta-toluamide (DEET), physician visits, diagnoses, and possible (self) treatment. To encompass incubation periods of DENV infection, participants made weekly diary entries from the first week of travel to two weeks after their return. Diaries were filled out on paper or digitally, and travelers received a weekly email as reminder. Blood samples for serologic testing were taken once during their pre-travel advice and once during a visit 2–6 weeks after return.

### Dengue-endemic countries

All (sub)tropical countries between the ten degrees January and July isotherm were considered dengue-endemic [13]. Although Spain, Italy, Turkey, United States, Japan, New Zealand, and Australia are countries (partially) within the ten degrees isotherms, these countries have good surveillance and therefore we know that there is no dengue risk or that the risk is limited to areas not frequently visited by travelers. Therefore, we considered these countries as “non-dengue-endemic country”. All countries outside the 10 degrees isotherms were also designated as “non-dengue-endemic country”.

### Laboratory methods

Blood samples were immediately stored at 6°C, then centrifuged and frozen at -80°C within 24 hours after collection. After all study participants had returned, all post-travel serum samples were thawed and tested for IgG antibodies to DENV antigen serotypes 1, 2, 3, and 4 by using an indirect ELISA (Panbio Diagnostics, Brisbane, Queensland, Australia) according to manufacturer’s instructions. For participants whose post-travel sample yielded positive test results, pre-travel samples were also tested for anti-DENV IgG.

The ELISA for anti-DENV IgG has a sensitivity of 90–100% and a specificity of 90–98% [14–16]. These test characteristics concern the use of paired serum samples. The presence of anti-DENV IgG in the pre-travel sample was considered suggestive of a previous DENV infection. The presence of anti-DENV IgG in the post-travel sample together with no anti-DENV IgG in the pre-travel sample was considered suggestive of acquiring a primary DENV infection during travel in the study period. We assumed that participants with a previous infection were still at risk for a secondary infection. Therefore, anti-DENV IgG in both the post- and pre-travel sample, but with a post-travel-to-pre-travel ratio of ≥4:1, was considered suggestive of a recent secondary DENV infection.

### Statistical analysis

To study the association between possible symptoms of a DENV infection and the presence of anti-DENV IgG, dengue-like illness (DLI) was defined as fever (temperature ≥38°C) with one of the following symptoms: myalgia, arthralgia, headache, retro-orbital pain, or skin rash [7, 8]. We defined ‘Extended DLI’ as DLI reported in ≥2 consecutive weeks. To study possible cross-reactivity of antibodies resulting from previous flavivirus vaccinations, we analyzed whether these vaccinations were predictive of the presence of anti-DENV IgG. For this purpose, we defined ‘vaccination status’ at the pre-travel visit as the total number of previously received yellow fever (YF), Japanese encephalitis (JE), and tick-borne encephalitis (TBE) vaccines. Vaccination status at the post-travel visit consisted of all additional YF/JE/TBE vaccinations received for the current travel. Due to small numbers of visiting participants per country, the visited countries were analyzed at continent level. If a traveler visited more than one continent, the continent in which the traveler spent most time was designated as the visited continent [17]. The use of DEET was quantified by dividing the number of weeks that the use of DEET was reported, by the number of weeks spent in dengue-endemic areas.

The following variables were examined as possible determinants of previous DENV infection at the pre-travel visit: sex, age, country of birth, total duration at previous travel destinations, and vaccination status (previously administered flavivirus vaccines). In the analysis of recent DENV infection, the following possible risk factors were examined: sex, age, country of birth, vaccination status (flavivirus vaccines administered before current travel), purpose of travel, (extended) DLI, and use of DEET.

Prevalence of previous DENV infection and the corresponding 95% confidence interval were calculated. We examined whether DENV prevalence differed by characteristics using univariable logistic regression. Variables with a p value <0.1 in univariable analysis were included in the multivariable model. The AR of recent DENV infection was calculated by dividing the number of study participants with a recent DENV infection by the total number of participants at risk. Incidence rate, incidence rate ratios (IRR), and 95% confidence intervals of recent DENV infections by potential risk factors were analyzed using poisson regression. Variables with a p value <0.1 in univariable analysis were included in the multivariable model. A p value <0.05 was considered statistically significant. As participants could have visited both dengue- and non-dengue-endemic countries, only the time spent in dengue-endemic countries was used as denominator to calculate DENV incidence rates. For those who became DENV infected while traveling, the moment of infection was estimated as the midpoint between the arrival and departure date in dengue-endemic countries, and therefore person-time denominators were divided in half. All analyses were conducted using STATA Intercooled version 13 (College Station, TX, USA).

## Results

### Characteristics of the study population

Between December 2008 and September 2011, 685 subjects who intended to travel to (sub)tropical countries for 13–52 weeks provided informed consent. Of these, 80 (12%) were excluded upon completion of the study: 42 had their travel arrangements changed, and 38 were lost to follow-up. We excluded another 4 individuals due to a missing pre- or post-travel blood sample, and one individual whose subtropical itinerary appeared not to include any dengue-endemic areas. We did not exclude three participants who traveled a few days less than 12 weeks, nor 5 participants who had traveled a few days over 52 weeks.

The median age of the 600 participants included in the present study was 25 years (interquartile range [IQR]: 23–29), 35.5% were male, 97.5% were born in a non-dengue-endemic country, median travel duration was 20 weeks (IQR: 15–25), and purpose of travel was predominantly tourism (62.2%) (Table 1). The median interval between return from travel and post-travel blood donation was 25 days (IQR 21–33). Fifty-five participants (9.2%) traveled to countries in ≥2 continents, of whom 24 participants were allocated as visitors to Asia, 24 to Latin America, and 7 to Africa. One participant visited Tonga (Oceania) exclusively and was counted as a visitor to Asia for simplicity purposes.

**Table 1:** Characteristics of 600 long-term travelers attending a Dutch travel health clinic for pre-travel advice including prevalence and determinants of previous dengue infection, December 2008 –September 2011.

Characteristic	Total, no	%	Previous DENV*		Univariable analysis				Multivariable analysis			
			No.	P %	OR	95% CI		p value	OR	95% CI		p value
						lower	upper			lower	upper	
No. Participants	600	100	19	3.2								
Sex								0.901				
Female	387	64.5	12	3.1	1							
Male	213	35.5	7	3.3	1.06	0.41	2.7					
Median age, y (IQR)	25 (23–29)				1.1	1.0	1.1	0.017				
Age, y								0.083				0.383
< 24	203	33.8	3	1.5	1				1			
24–29	261	43.5	8	3.1	2.1	0.55	8.0		1.8	0.47	7.1	
≥ 30	136	22.7	8	5.9	4.2	1.1	16.0		2.6	0.63	10.9	
Country of birth								0.491				
Non-dengue-endemic country	585	97.5	18	3.1	1							
Dengue-endemic country	15	2.5	1	6.7	2.3	0.28	18.1					
Total duration at previous travel destinations, mth								0.006				0.025
< 1	263	43.8	2	0.76	1				1			
1–3	114	19.0	6	5.3	7.3	1.4	36.5		6.6	1.3	33.6	
> 3	223	37.2	11	4.9	6.8	1.5	30.9		5.0	1.0	24.6	
Vaccination status: total of pre-travel YF/JE/TBE** vaccinations								0.221				
0	377	62.8	9	2.4	1							
1	202	33.7	8	4.0	1.7	0.64	4.4					
≥ 2	21	3.5	2	9.5	4.3	0.87	21.3					

* DENV = Dengue virus.

** YF = yellow fever, JE = Japanese encephalitis, TBE = tick-borne encephalitis

### Vaccination status

Before inclusion, 223 participants (37%) had received one or more flavivirus vaccinations: 217 travelers (36%) had received ≥1 prior yellow fever vaccination, 8 (1%) had ≥1 Japanese encephalitis vaccination, and 4 (1%) ≥1 tick-borne encephalitis vaccination. For the current trip, 177(30%) received ≥1 YF/JE and/or TBE vaccinations, including 18 previously vaccinated individuals. Of these 177, 8 participants received ≥2 vaccinations: all 2 or 3 JE vaccinations for a trip to Asia.

### Previous DENV infection

Anti-DENV IgG in the pre-travel sample suggestive of previous DENV infection were found in 19/600 participants (3.2%; 95% CI 17.6–45.7) (Table 1). In univariable logistic regression analysis, previous DENV infection was associated with older age and longer duration of previous travels to (sub)tropical regions, but not with vaccination status, gender, and country of birth (yes/no dengue-endemic country). In multivariable analysis, only previous duration of travel remained significantly associated with a previous DENV infection.

### Recent DENV infection

In 39 of 600 participants the anti-DENV IgG test results were suggestive of recent DENV infection (AR = 6.5%; 95% CI 4.5–8.5); the IR was 13.7 per 1,000 person-months (95% CI 9.8–18.8) (Table 2). The attack rate was 4.2% (7/166) for those traveling 12–15 weeks, 5.7% (9/158) for those traveling 16–20 weeks, 8.9% (13/146) for those traveling 21–25 weeks and 7.7% (10/130) for those traveling ≥26 weeks.

**Table 2:** Characteristics of 600 long-term travelers to dengue-endemic areas attending a Dutch travel health clinic for pre-travel advice including their incidence rates and risk factors of suggestive recent dengue virus infection, December 2008 –September 2011.

Characteristics	Total			Recent DENV*		Univariable analysis				Multivariable analysis			
	No.	%	Person-months	No.	IR/1000 pm	IRR	95% CI		p value	IRR	95% CI		p value
							lower	upper			lower	upper	
No. participants	600	100	2796.0	39	13.7								
Sex									0.412				
Female	387	64.5	1756.6	22	12.5	1							
Male	213	35.5	1039.5	17	16.4	1.3	0.69	2.5					
Age, y									0.285				
< 24	203	33.8	883.7	10	11.3	1							
24–29	261	43.5	1225.0	15	12.2	1.1	0.49	2.4					
≥ 30	136	22.7	687.4	14	20.4	1.8	0.80	4.1					
Purpose of travel													
Tourism	373	62.2	1656.3	27	16.3	1			0.333				
Work/education	172	28.7	849.6	10	11.8	0.72	0.35	1.5					
VFR/other	55	9.2	290.1	2	6.9	0.42	0.10	1.8					
Visited continents									0.926				
Asia	268	44.7	1187.2	16	13.5	1							
Africa	107	17.8	505.2	8	15.8	1.2	0.50	2.7					
Latin America	225	37.5	1103.7	15	13.6	1.0	0.50	2.0					
Vaccination status: all additional YF/JE/TBE** vaccinations for current trip†									0.027				0.020
0	423	70.5	1967.0	24	12.2	1				1			
1	169	28.2	799.2	12	15.0	1.2	0.62	2.4		1.3	0.64	2.6	
≥ 2	8	1.3	29.8	3	100.6	8.2	2.5	27.4		9.3	2.8	31.1	
Use of DEET***, percentage of total travel duration									0.932				
< 25	173	28.8	869.9	12	13.8	1							
25–49	124	20.7	559.8	9	16.1	1.2	0.49	2.8					
50–74	111	18.5	525.5	6	11.4	0.83	0.31	2.2					
≥ 75	192	32.0	840.8	12	14.3	1.0	0.46	2.3					
Dengue-like illness^									0.196				
No	422	70.3	1923.9	23	12.0	1							
Yes	178	29.7	872.1	16	18.3	1.5	0.81	2.9					
Extended dengue-like illness^^									0.041				0.028
No	574	95.7	2668.1	34	12.7	1				1			
Yes	26	4.3	127.9	5	39.1	3.1	1.2	7.8		3.4	1.3	8.8	

* DENV = Dengue virus.

** YF = yellow fever, JE = Japanese encephalitis, TBE = tick-borne encephalitis.

*** DEET = N,N-diethyl-meta-toluamide. The use of DEET was quantified by dividing the number of weeks that the use of DEET was reported, by the number of weeks spent in dengue-endemic areas.

^†^ Vaccination status in this table includes all additional flavivirus vaccines received from pre-travel until post-travel visit.

^ DLI was defined as fever (temperature ≥38°C) with one of the following symptoms: myalgia, arthralgia, headache, retro-orbital pain or skin rash. In this table, DLI was considered positive if a traveler reported DLI in ≥ 1 week.

^^ ‘Extended DLI’ includes all travelers who reported at least DLI in ≥2 consecutive weeks.

**Fig 1:**
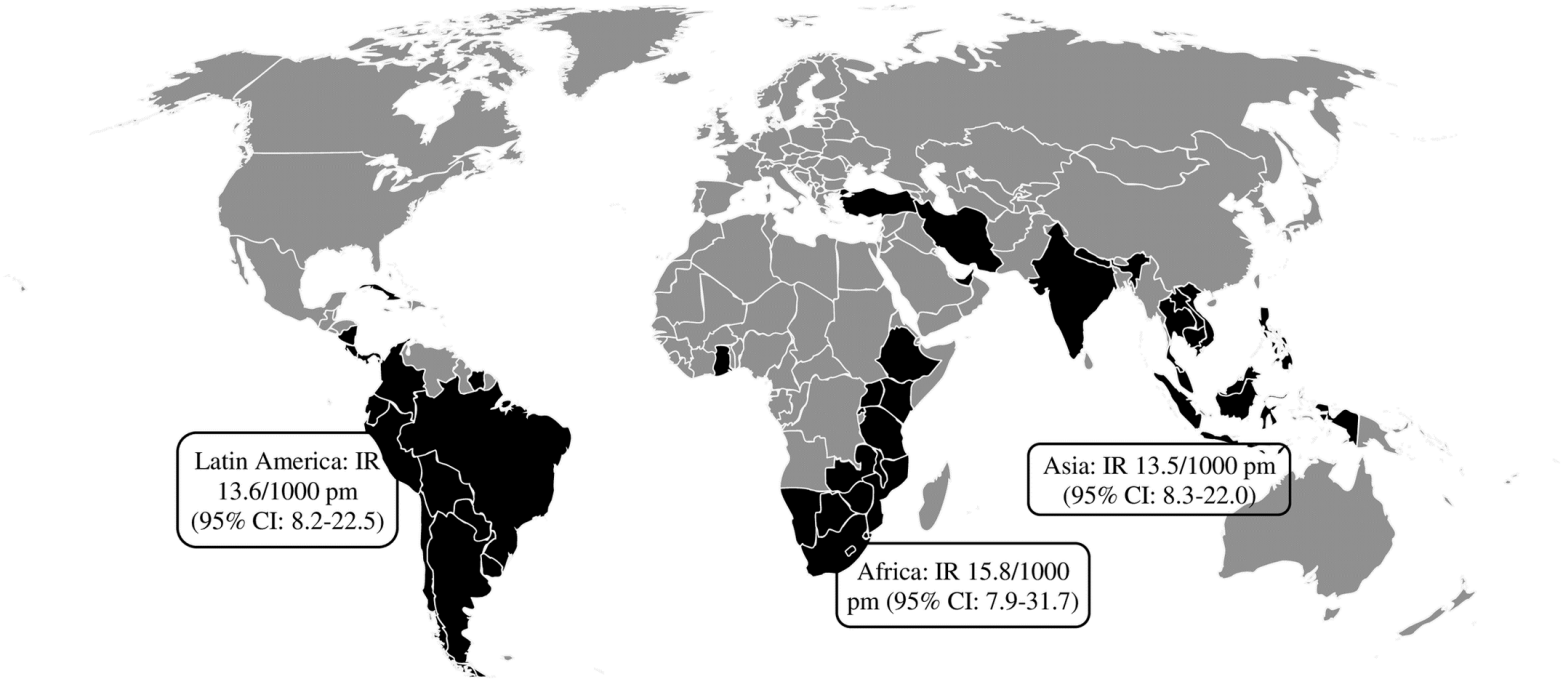
World map: Countries visited by the participants with evidence suggestive of recent dengue virus infection. Reprinted from LCR under a CC BY license, with permission from LCR, original copyright 2017.

The median age of the 39 participants with recent DENV infection was 27 years (IQR: 23–35), 17 (44%) subjects were male, and median total travel duration was 22 weeks (IQR: 17–26). All 39 participants were born in a non-endemic country. One participant with a recent DENV infection traveled in both Latin America and Asia. As he spent most days in dengue-endemic areas in Latin America, he was categorized as a traveler to Latin America, making the incidence rate of recent DENV infection 13.5 (95% CI: 8.3–22.0), 15.8 (95% CI: 7.9–31.7), and 13.6 (95% CI: 8.2–22.5) for Asia, Africa, and Latin America respectively (Fig 1). None of the 39 travelers with recent DENV infection had evidence of anti-dengue antibodies in the pre-travel sample suggesting they all had a primary DENV infection.

In total, DLI was reported in 262 weeks by 178/600 (30%) participants. Among the 39 participants with recent DENV infection, DLI was reported by 16 (41%) travelers (29 weeks in total) and included 5 participants with a period of extended DLI. Three travelers with recent DENV infection (8%) reported fever during travel but did not meet the DLI criteria, and the other 20 travelers with a recent DENV infection (51%) did not report any episodes of fever at all. Of all travelers, 26/600 (4.3%) participants were hospitalized, including 4 of the 39 travelers (10%) with recent DENV infection. All four were hospitalized during a period of extended DLI, but none of them reported signs or symptoms of a hemorrhagic fever. Whereas DLI was not predictive of the presence of anti-DENV IgG in the post-travel sample in the univariable analysis, the effect of extended DLI was significantly increased (IRR 3.1, 95% CI 1.2–7.8) compared to participants without extended DLI.

Vaccination status (all additional YF/JE/TBE vaccinations received for the current trip) was also predictive of recent DENV infection; the IRR was significantly higher for participants who received 2 or more vaccinations compared to those who did not receive additional vaccinations (IRR 8.2, 95% CI 2.5–27.4).

The effect of sex, age, purpose of travel, visited continents, and use of DEET was not significant. In the multivariable model, both extended DLI and the vaccination status remained significantly related to a recent DENV infection.

Our analysis sugggested that the 3 recent DENV infections among participants who had received 2 or more JE-vaccinations for a trip to Asia, could be false-positive due to cross reaction caused by these vaccinations. Therefore, we recalculated the attack and incidence rate without these cases. The AR was now 6.0% (36/597), the IR was 12.9/1,000 person-months (95% CI 9.1–17.9), and the IR_Asia_ decreased to 11.0/1,000 person-months (95% CI 6.4–18.9). Vaccination status (all additional YF/JE/TBE vaccinations received for the current trip) was now no longer predictive of a recent DENV infection in univariable analysis; the IRR was comparable for participants who received 1 or more vaccinations compared to those who did not receive additional vaccinations (IRR 1.2, 95% CI 0.59–2.3, p = 0.648). Only extended DLI remained significantly related to a recent DENV infection.

## Discussion

In this prospective study of long-term travelers to (sub)tropical countries we found a substantial AR and IR for DENV infection during travel. Our estimated AR ranged from 6.0% to 6.5% and is higher than found in previous prospective studies among short-term travelers in which ARs ranged between 1% and 2.9% [7–10]. The only other prospective study among long-term travelers (3–6 months) found a comparable AR of 6.7% [11]. As expected, the duration of travel influences the AR, while it does not influence the incidence rate. Hence, unlike the AR, the DENV incidence observed in our study was comparable to those reported in other prospective studies, which varied from 6.7 to 30 per 1000 person-months among the predominantly short-term travelers, and was 11 per 1000 person-months among the long-term travelers [7–11]. Comparison of results between different prospective studies should, however, be interpreted with caution. The risk for travel-related DENV infection depends not only on endemicity, but also on outbreaks in a particular country during a particular time of travel. As the journeys of the participants were scattered over countries and over time, the influence of possible rain seasons or outbreaks in this cohort of long-term travelers could not be studied. Also, comparison of prospective studies with studies based on surveillance data of travelers returning ill should be done with greatest caution: increased awareness of dengue in hospitals and improvements in diagnostic procedures could also have influenced the increase of diagnosed DENV infections in surveillance data [5].

Similar to previous prospective studies, we did not find differences between IRs among travelers to Asia, Latin America, and Africa [7, 9, 11]. Therefore, it is remarkable that dengue is still diagnosed relatively less often in febrile returning travelers from Africa compared to Asia and Latin America [5]. DENV diagnosis may be missed in patients returning from Africa, as it is not considered in the differential diagnosis.

In our study, 51% of the participants with a recent DENV infection did not report any episode of fever suggesting an asymptomatic infection. On the other hand, 16/39 of the travelers with test results suggestive of a DENV infection during their trip reported DLI at least once, 5 of whom reported extended DLI. Although DLI was not related to a recent DENV infection, participants who reported extended DLI were more likely to have a recent DENV infection. Possibly because DLI includes common and aspecific symptoms like headaches, brief episodes of DLI are mostly caused by other infections with fever, whereas extended and more severe symptoms of DLI are more specific for a recent DENV infection. The fact that all four hospitalizations among the travelers with a recent DENV infection occurred in the 5 participants with an extended DLI episode supports this hypothesis. Considering the possible severity of the disease, it is still important that travelers are advised pre-travel to take proper anti-mosquito measures, especially as no dengue vaccine is yet available for travelers.

Selection bias may have influenced risk estimates found in studies among cohorts of travelers, including ours. First, travelers often choose to avoid areas if an outbreak is reported, in contrast to people living in endemic regions. This could partly explain why incidence rates of DENV virus infection among travelers seems to have remained rather similar in the past two decades, despite an increase in reported cases among populations in endemic countries. Furthermore; participants who seek pre-travel health advice may have higher than average health awareness, particularly once they learn about the study, agree to participate, and keep a diary during travel. This could have led to an underestimation of the true incidence of DENV infections.

Although travelers who had received one flavivirus vaccination for the current trip were not more likely to acquire a recent DENV infection than travelers who did not receive any additional flavivirus vaccines, the 8 travelers who received 2 or more flavivirus vaccines were significantly more likely to acquire a recent DENV infection. As all of these eight participants had received JE vaccines (vs. 2 out of 169 participants who had 1 additional JE vaccine), cross-reaction between JE and anti-DENV IgG cannot be excluded. However, when the potential false-positive cases were excluded, the AR and IR only decreased slightly. Therefore, at most, it could be considered of limited influence on the outcome of this study. Although not significantly, Asia’s IR became notably lower than both the IRs of Africa and Latin America after excluding the potential false positives. This was somewhat surprising as a previous review article concluded that most cases of dengue infections in febrile travelers were diagnosed among travelers from Asia [18]. It reinforces our conclusions that some of these cases were possibly true infections and other unmeasured confounders (e.g., the kind of travel (low budget/adventurous) or infection with yet another flavivirus) could also have been of influence, as all participants with ≥2 JE vaccines traveled to Asia.

Thus far no true standard exists to serologically confirm or rule out dengue after an infection. We considered testing anti-DENV IgM as well as anti-DENV IgG in our cohort, but argued this would have been of limited added value for our group, as all participants had been traveling for a substantial period and most of them donated their post-travel blood sample at least two weeks after return. We therefore assumed it to be highly unlikely for travelers to contract DENV in their last days of travel. As a consequence, positive post-travel IgM results without any detectable IgG-levels would have been unlikely, and waning IgM-levels could have led to negative post-travel results. The use of a plaque-reduction neutralization test, which is considered the laboratory standard, was not part of the study protocol, but could also have cross-reacted with other flavivirus antibodies [19]. However, the study was performed before the large Zika virus outbreaks in the Americas; therefore cross-reaction with Zika is probably of little influence. Furthermore, we found no significant relation between flavivirus vaccines and the presence of anti-DENV antibodies in previous serology-based studies [7, 20].

Finally, some caution is required in the interpretation of our data, as data collection happened through self-reported weekly diaries. Participants could have interpreted the weekly questions differently. For example, some may have ticked ‘used of DEET’ if they used DEET at least once, others may have ticked itif they used DEET every day that specific week. This might have influenced the results regarding ‘use of DEET’ as a predictor for DENV infection. However, as diaries decrease the effect of recall bias, we do not consider this limitation to have significantly affected other findings.

## Conclusion

This is the second prospective study investigating DENV infection among long-term travelers. It confirms that the incidence rate of DENV infection among long-term travelers is substantial. As expected, the attack rate was higher among long-term than among previously investigated short-term travelers. Half of the travelers with a recent DENV infection reported no symptoms of dengue-like illness, suggesting they had asymptomatic infections, but almost all DENV-infected travelers who reported DLI symptoms in >1 consecutive weeks were hospitalized.

## Supporting information

S1 File Permission form for Fig 1. (PDF)

S1 Dataset Dataset long-term travelers (de-identified). (XLSX)
