# Supplementary material for: Dengue virus infection among long-term travelers from the Netherlands: A prospective study, 2008-2011
Source: PLoS One. 2018 Feb 7;13(2):e0192193. doi: 10.1371/journal.pone.0192193 (PMC5802908; doi:10.1371/journal.pone.0192193)
Supplement: S1 File — (PDF) [file pone.0192193.s001.pdf]

To: Mr GJB Sonder
LCR
Nieuwe Achtergracht 100
1018 WT Amsterdam
The Netherlands

From: Femke Overbosch
GGD Amsterdam
Nieuwe Achtergracht 100
1018 WT Amsterdam
The Netherlands

Subject: Permission to use figure

4 January 2018

Dear Mr Sonder,

I request permission for the open-access journal PLOS ONE to publish 'the world map' of the LCR in our submitted article 'Dengue virus infection among long-term travelers from the Netherlands; a prospective study, 2008-2011'under the Creative Commons Attribution License (CCAL) CC BY 4.0 (http://creativecommons.org/licenses/by/4.0/).

Please be aware that this license allows unrestricted use and distribution, even commercially, by third parties.

Please reply by completing next paragraph in which you provide explicit written permission to publish the world map under a CC BY license."

I provide permission to publish figure 1

....

....

Signature:.. Date: 9 January 2018

g.j.b. sonder, arts

Director LCR

Kind regards,

~~Femke Overbosch~~
